# Principal components analysis and the reported low intrinsic dimensionality of gene expression microarray data

**DOI:** 10.1038/srep25696

**Published:** 2016-06-02

**Authors:** Michael Lenz, Franz-Josef Müller, Martin Zenke, Andreas Schuppert

**Affiliations:** 1 Joint Research Center for Computational Biomedicine, RWTH Aachen University, 52062 Aachen, Germany; 2 Aachen Institute for Advanced Study in Computational Engineering Science (AICES), RWTH Aachen University, 52062 Aachen, Germany; 3 Maastricht Centre for Systems Biology (MaCSBio), Maastricht University, 6229 ER Maastricht, Netherlands; 4 Zentrum für Integrative Psychiatrie, University Hospital Schleswig-Holstein, Campus Kiel, Kiel, Germany; 5 Institute for Biomedical Engineering, Department of Cell Biology, RWTH Aachen University Medical School, 52074 Aachen, Germany; 6 Helmholtz-Institute for Biomedical Engineering, RWTH Aachen University, 52074 Aachen, Germany

## Abstract

Principal components analysis (PCA) is a common unsupervised method for the analysis of gene expression microarray data, providing information on the overall structure of the analyzed dataset. In the recent years, it has been applied to very large datasets involving many different tissues and cell types, in order to create a low dimensional global map of human gene expression. Here, we reevaluate this approach and show that the linear intrinsic dimensionality of this global map is higher than previously reported. Furthermore, we analyze in which cases PCA fails to detect biologically relevant information and point the reader to methods that overcome these limitations. Our results refine the current understanding of the overall structure of gene expression spaces and show that PCA critically depends on the effect size of the biological signal as well as on the fraction of samples containing this signal.

Principal components analysis (PCA) is frequently used in biological sciences for global analysis of omics datasets. It provides fully unsupervised information on the dominant directions of highest variability in the data and can therefore be used to investigate similarities between individual samples, or formation of clusters^1^. These unsupervised similarity information is then usually compared to data annotations or some phenotypic information to detect previously unknown relationships or for characterization of samples that lack proper phenotypical annotation.

In most of these studies, scientists focus on the first two to four principal components (PCs), assuming that higher-order components mainly contain irrelevant information or noise^2^. This assumption is confirmed by a few studies investigating this topic in a systematic way. One study performed by Schneckener *et al*.^3^, states that gene expression differences that are represented in higher than the first four PCs are rarely reproducible in new experiments.

Another study by Lukk *et al*.^4^ also suggests a surprisingly low dimensionality of gene expression data. They performed PCA and hierarchical clustering on a larger and heterogeneous microarray dataset (5372 samples from 369 cell types, tissues, or disease states) and found that the first three PCs have clear biological interpretations. The fourth component already correlates with an array quality metric and thus represents measurement noise. The hierarchical clustering analysis supported these results, detecting the same major clusters that were found in the PCA.

Finally, there is also some evidence from wet-lab biology that gene expression of a distinct cell type might be mainly controlled by only a few independent factors, due to the possibility to reprogram various adult somatic cell types by exogeneous expression of only four transcription factors into induced pluripotent stem cells^5^.

While this low-dimensionality in gene expression seems to be a common and prevalent observation, we show here that there are more than three to four biologically relevant linear dimensions in large heterogeneous gene expression datasets. Furthermore, we explain in which cases PCA fails to detect relevant embedded information and suggest methods that could complement PCA for a more comprehensive detection of underlying genetic or non-genetic variability in large-scale gene expression compendia.

## Results

### PCA on large heterogeneous microarray gene expression datasets

We first reproduced the results from Lukk *et al*.^4^, performing PCA on their dataset consisting of 5372 samples from 369 different tissues, cell lines, or disease states that were hybridized on the Affymetrix Human U133A microarray. The first three PCs of this analysis are associated with hematopoietic cells, malignancy (mainly proliferation), and neural tissues, respectively (Supplementary Fig. S1a, reproduced from^4^). The fourth PC does not correlate with any biological annotation, but with an array quality metric (relative log expression, RLE). Thus, it was assumed that this PC essentially represents measurement noise. Schmid *et al*.^6^ report slightly different results based on a smaller dataset with 3030 samples. The authors could confirm a separation of hematopoietic and neural tissues from all others on their first two principal components, but did not identify a PC separating cell line from tissue samples (although this separation may have been observable in higher PCs, which were not included in the paper).

**Figure 1:**
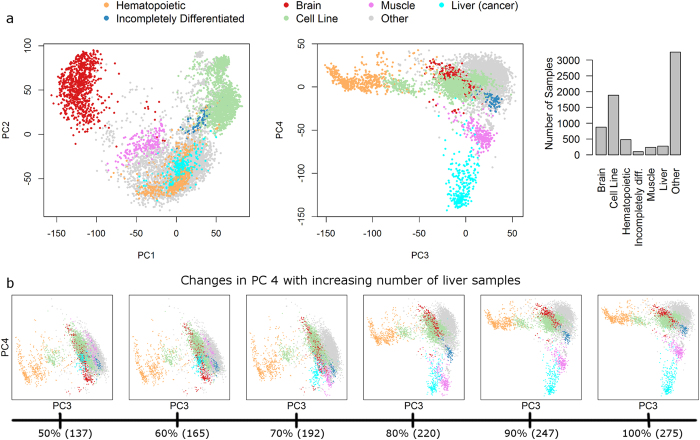
PCA based analysis of the large scale structure in gene expression data. (**a**) PCA was applied to the own dataset, replicating the separation of brain tissues, cell lines, and hematopoietic tissues from all others in the first three PCs as reported by Lukk *et al*., but revealing different orientations of these three PCs. The fourth PC is associated with liver and hepatocellular carcinoma samples, in stark contrast to the noise association reported by Lukk *et al*. Details of the classification into the 7 color coded groups are given in Supplemental Table S2. The sample distribution among these groups differs (**b**) Detection of a liver-specific signal in PC 4 depends critically on the number of liver (cancer) samples included in the analyzed dataset. A reduction of the number of liver (cancer) samples to 50 or 60% completely erases any liver-specificity of PC 4. This explains the observed difference in PC 4 between the Lukk dataset and our own dataset, since the proportion of liver (cancer) samples in the Lukk dataset is only 30% of that in the own dataset.

We assembled an additional larger dataset consisting of 7100 samples from the Affymetrix Human U133 Plus 2.0 microarray platform in order to compare the reported results to those obtained with a different dataset. Applying PCA to this dataset replicates the separation of hematopoietic cells, cell lines, and neural tissues from all other cells on the first three PCs (Fig. 1a) as reported by Lukk *et al*.^4^. However, the fourth PC of our dataset separates liver and hepatocellular carcinoma samples from all others and the first occurrence of a PC without clear biological interpretation is in component 5 (Supplementary Fig. S2).

Thus, the first three PCs derived by Lukk and our own dataset seem to span similar spaces by separating samples mainly in hematopoietic, brain, and cell line related groups, although with rotated coordinate axes, while further components differ more strongly between the two datasets. In order to quantify the similarities and differences between our dataset specific PCA model and the model published by Lukk *et al*., we employed a linear regression approach comparing each of the first three PCs of our PCA model using the first three PCs derived from the Lukk dataset. This results in surprisingly high R-squared values of 0.74, 0.66, and 0.70, indicating a strong concordance of our first three PCs with those reported by Lukk *et al*. In contrast, trying to match the fourth PC of the own dataset, which captures the liver dimension specific to our dataset, by the first 5 PCs of the Lukk dataset results in a non-significant R-squared value of 0.07. This measure can be increased to a still comparably low value of 0.50 when the first 10 PCs of the Lukk dataset are combined as regressors. Thus, the liver-specific component in our own dataset is incompletely represented in the first 10 PCs of the Lukk dataset.

Next, we set out to explore how the observed differences in the PCAs of the two datasets could be explained and assumed that different sample numbers for the various cell types in the respective datasets could have a major effect. The Lukk *et al*.^4^ dataset contains a very large amount of hematopoietic samples (Supplementary Fig 1a), which might be responsible for their separation in the first PC, while our own dataset incorporates a comparably high number of liver and hepatocellular carcinoma samples (3.9% of all samples as opposed to 1.2% in the Lukk dataset), potentially explaining the direction of the fourth PC.

To test this hypothesis, we performed two computational experiments. First, we downsampled our dataset (consisting of 7100 samples) to a specifically selected subset of 1394 samples in order to obtain a similar sample distribution as in the Lukk dataset in terms of the proportion of samples from the hematopoietic system, brain, cell lines, muscle, and incompletely differentiated cells (Supplementary Fig. S1b). We then performed a PCA on this downsampled dataset and found a surprisingly high similarity to the model reported by Lukk *et al*, revealing almost identical patterns of sample separation in PCs 1–3 (Supplementary Fig. S1b).

As a second computational experiment, we went back to the full dataset of 7100 samples and varied only the number of liver (cancer) samples, exploring corresponding changes in the liver-specific PC 4. This analysis reveals that the direction of PC 4 changes significantly with increasing number of liver (cancer) samples (Fig. 1b). When the number of liver (cancer) samples is reduced to 60% or less of the original 275 liver (cancer) samples, no clear biological interpretation can be observed in PC 4 anymore. This corresponds to the case of the Lukk dataset, where the proportion of liver samples (1.2%) is only roughly 30% of the proportion of liver samples in our own dataset (3.9%). An increase of the number of liver samples to 70% or 80% leads to a joint separation of liver and muscle samples on PC 4. A further increase then rotates the direction of PC 4 slowly away from muscle samples, more towards the direction of the liver samples. Together, these two computational experiments validate our hypothesis on sample size effects in PCA, explaining the apparent differences of the two original PCA models.

### Information content beyond the first three PCs

In both, the Lukk and our own dataset, the first three PCs explain roughly 36% of the variability. The remaining 64% contain partly noise, but we were interested if there is also important biological information contained in these components.

In order to investigate the information content in higher PCs, we decomposed the original dataset into two derived datasets with the same number of genes and samples as the original dataset. The “projected” dataset captures the information in the first three PCs, while the “residual” dataset represents the information in the fourth and all higher PCs.

**Figure 2:**
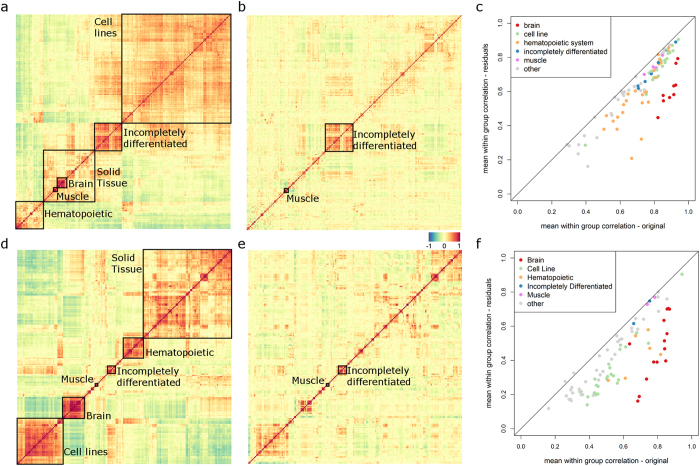
Inter- and intra-tissue correlation before and after PCA-based decomposition. (**a,d**) Correlation matrix of the 369 (**a**) or 192 (**d**) tissue specific expression patterns derived from the Lukk dataset (**a**) or the own dataset (**d**) shows strong correlations between the signatures of the large scale groups (boxes and text). The ordering is based on hierarchical clustering of the columns and rows (euclidean distance, complete linkage). (**b,e**) Residual tissue specific signatures of the Lukk (**b**) or own (**e**) datasets after PCA-based decomposition show strongly reduced correlations between each other. Only very few clusters remain in comparison to (**a**,**d**). The ordering of columns and rows in (**b,e**) is the same as in (**a,d**) respectively. (**c,f**) In contrast to the strong reduction of between-signature correlations, correlations between samples of the same group, i.e. within a specific tissue or cell type, remain high. Shown are the mean within-group correlations of all groups that have at least 10 samples before (abscissa) and after (ordinate) PCA-based decomposition. This is a first indication that there is significant tissue specific information in the residual space.

In a first analysis step, we compare the correlation between the genome-wide expression in different tissues in the original datasets (Fig. 2a,d) to that in the residual datasets (Fig. 2b,e). This analysis shows a strong overall reduction of correlation patterns in the residual dataset, suggesting that the first three PCs cover large amounts of inter-tissue correlation. In contrast, most tissue specific information seems to remain in the residual space after subtraction of the first three PCs, as indicated by the still relatively high correlation between samples from the same tissue in the residual space (Fig. 2c,f). This observation seemingly contradicts the widely held hypothesis that most relevant information is contained in the first three PCs^2^, as one would expect that the correlations within a specific group of samples would reduce to a value close to zero.

**Figure 3:**
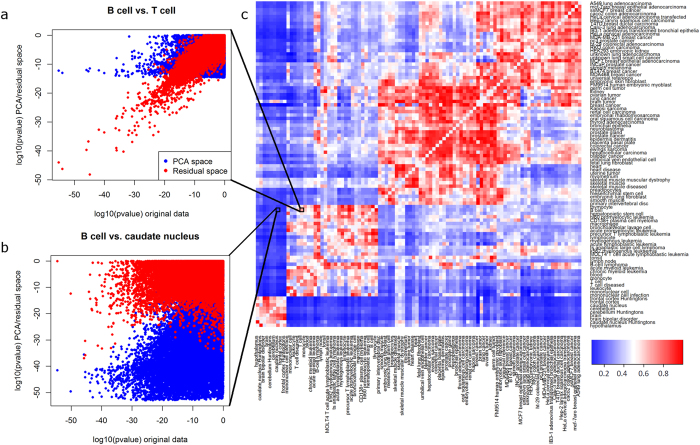
Information content in the PCA-space and the residual space measured by log-10 p-values. (**a,b**) The information partition between the projected space and the residual space is shown for 2 different comparisons, validating the high tissue-specific information in the residual space (strongly negative p-values). Blue (red) points depict the log-10 p-value in the projected space (the residual space) in comparison to the log-10 p-value in the original dataset. The corresponding IR values (Schneckener *et al*.^3^) of the 2 comparisons are 0.7103 (**a**), and 0.1641 (**b**). (**c**) Heatmap of IR values for all pairwise comparisons of the 96 groups with at least 10 samples in the Lukk dataset. Blue colors correspond to low IR values, indicating that most information is in the PCA space, while red colors correspond to high IR values, meaning that the residual space contains more information than the PCA space. In general, it can be observed that within one of the large scale groups the IR is very high (**a,c**), while it is rather low for comparisons between tissues from different large scale groups (**b,c**).

In order to formalize the measurement of tissue-specific information content in the residual space, we utilize a previously developed information ratio criterion^3^. The information ratio (IR) is a means to quantify the distribution of information content between the projected (here: the first three PCs) versus the residual gene expression space (here: gene expression after subtracting the first three PCs). The IR uses genome-wide log-p-values of gene expression differences between two phenotypes to measure the amount of phenotype-specific information in the residual space compared to the projected space (Materials and Methods). Application of the IR to pairwise comparisons of groups that have at least 10 samples in the Lukk dataset confirms that there remains significant relevant information in the residual space (Fig. 3). Especially for comparisons within the large scale groups, e.g. between two brain regions, two hematopoietic cell types, or two cell lines, most of the information is contained in the residual space, while for comparisons between tissues from different large scale groups a relevant amount of information resides in the projected space (Fig. 3). These results corroborate our correlation analysis results (Fig. 2), indicating that tissue-specific information is contained in the residual space, suggesting that the linear dimensionality of gene expression spaces may be higher than has been suggested before^4^.

### Analysis of sample subsets reveals further information

We could already show that PCA depends strongly on the specific choice of samples in the dataset, which is why we set out to investigate whether we can detect biologically relevant information in the residual space by applying PCA on a subset of the Lukk dataset. Therefore, we used two different subsets based on the biological annotation of the dataset: A “brain subset” consisting of all brain samples in the Lukk dataset, which we further subdivide according to the brain region into samples from caudate nucleus, hypothalamus, frontal cortex, and cerebellum and a “cancer subset” consisting of all colorectal cancer, ovarian cancer, and hepatocellular carcinoma samples in the Lukk dataset.

**Figure 4:**
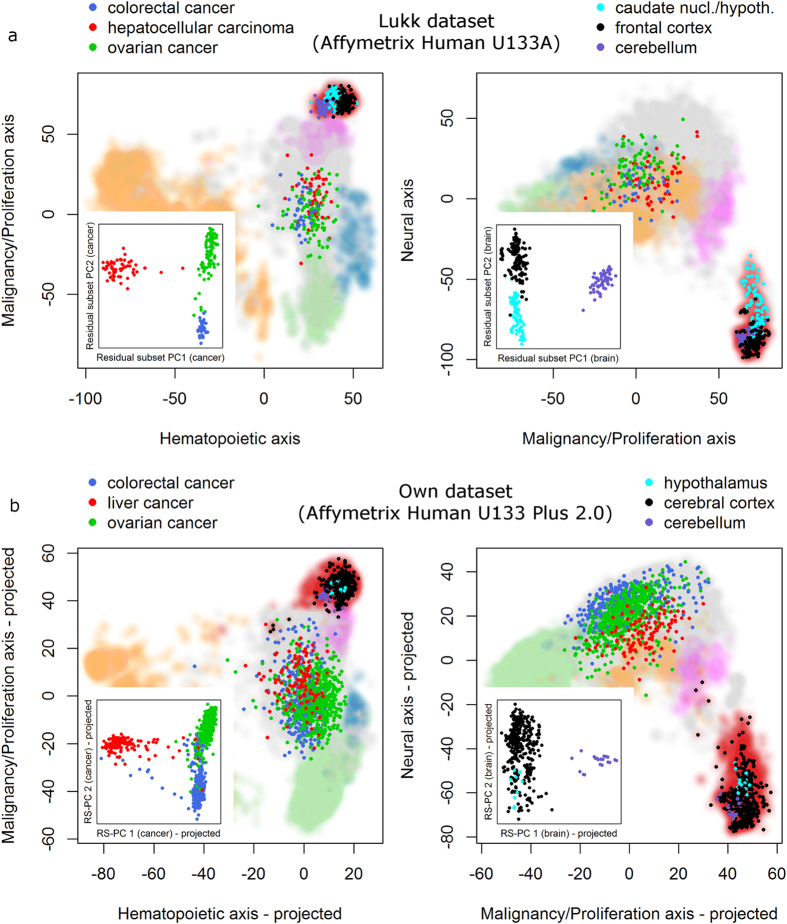
PCA on subsets of data can reveal additional dimensions. (**a**) Two different subsets of the Lukk dataset, a brain subset and a cancer subset, were analyzed in more detail. The larger graphics show their location on the first three PCs of the complete dataset with color coding according to cancer primary site or brain region. The inset (small graphics) show the first two PCs (called “residual subset PCs”) of a PCA applied to the residual data matrix of the cancer (left) or brain (right) subset. The analysis shows that the different cancers or brain regions can be nicely separated on the residual space while they overlap more strongly on the three dimensional PCA space derived based on the complete dataset. (**b**) The dimensions derived in (**a**) can be projected to the own dataset, i.e. across microarray platforms, showing a similar separation of cancer types and brain regions on the residual space. This verifies the actual biological relevance of the additional dimensions in the residual space (insets). Background colors represent the complete Lukk dataset (**a**) or own dataset (**b**) with colors according to large-scale groups (red: brain, orange: hematopoietic, green: cell line, blue: incompletely differentiated, magenta: muscle, grey: other).

As a first step, we visualized the two subsets in the three dimensional PCA space published by Lukk *et al*. (Fig. 4a), i.e. we did not do a separate analysis on the subsets yet, but just visualized the location of the two subsets within the PCA results from the complete Lukk dataset. This reveals a relatively good distinction of the different brain regions on the second and third PCs, whereas the three different cancer types overlap strongly on all three PCs, indicating that there is no information on the different cancer types within the first three PCs of the entire dataset. We then concentrated our analysis on the residual space and performed PCA separately on the two subsets after subtracting out the information from the first three Lukk-PCs. This analysis reveals a very clear distinction of the different brain regions as well as the different cancer types in the first two “residual subset PCs” of the respective subset analyses (Fig. 4a insets). Thus, this indicates again that there is relevant information beyond the first three PCs, since we performed this subset analysis on the residual dataset, i.e. with the information of the first three Lukk-PCs subtracted out. Notably, the distinction of the three cancer types was not detectable in the first three PCs of the overall dataset, meaning that the complete information about the differences in these cancers is contained in the residual space. The difference between the different brain regions, in contrast, is partly contained in the projected space and partly in the residual space. The reason for detecting additional biological information in the subset analyses is that the proportion of samples representing this biological information is increased in the subsets (due to a lower overall sample size). This increases the proportion of explained variance of these biological signals (see Supplemental Discussion 1).

One potential shortcoming of the analysis performed so far is that the large dataset we are dealing with is composed of data from many different studies. Therefore, one might argue that the detected information in the residual space, i.e. the “residual subset PCs”, represents mainly study-specific effects and is not reproducible. In order to exclude this possibility, we use our own dataset as an independent validation dataset to confirm the biological relevance of the residual subset PCs. We first projected our complete dataset on the three PCA axes from the Lukk dataset, revealing the same distinction of the different large scale groups as reported by Lukk *et al*. (Supplemental Fig. S3). This shows that these axes can be stably projected to a different Affymetrix array. We then focused on the two subsets and identified the corresponding samples of the three different cancer types and different brain regions in the own dataset. A visualization of the brain subset on the three projected axes derived from Lukk *et al*. showed a less clear distinction of the different brain regions on the PCA space than detected in the Lukk dataset before (Fig. 4b compared to Fig. 4a). The different cancer types showed again a strong overlap on these three projected PCA-dimensions, in great similarity to the results on the Lukk dataset.

We then again looked at the residual space (after subtracting out the three projected Lukk-dimensions) and projected these residual subsets from the own dataset on the “residual subset PCs” detected based on the corresponding subset from the Lukk dataset before. This reveals a distinct separation of the three cancer subtypes as well as the cerebellum samples, while the hypothalamus samples showed a less clear distinction from cerebral cortex than before (Fig. 4b insets). This analysis validates the reproducibility of the detected “residual subset PCs”, clearly demonstrating that there is indeed biologically relevant information beyond the first three PCs that can be detected by subset analyses.

### Two cases in which PCA fails to detect biologically relevant information

Having shown that there can be important information that is not properly found by performing a standard PCA on the complete dataset, we investigated more theoretically (see Supplemental Discussion 1) in which cases this failure of detecting important biological information occurs and suggest methods to overcome this shortcoming. Based on an illustrative statistical model (see Supplemental Discussion 1) we identified two structurally different cases in which biological information is not detected by PCA even under linear assumptions.

**Figure 5:**
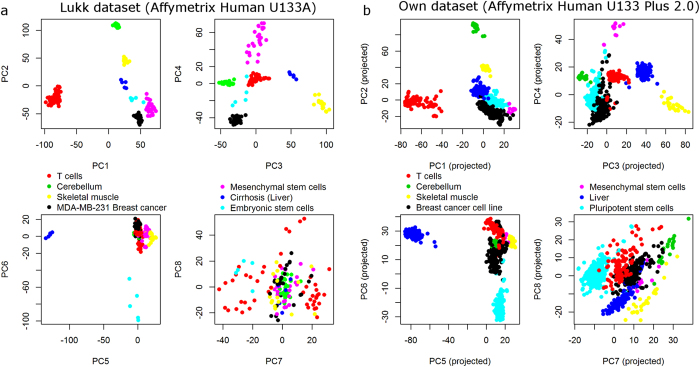
Specific sample choices can result in more (here 6) biologically interpretable principal components. PCA was performed on a subset of the Lukk dataset (**a**) resulting in 6 components with clear biological meaning. These 6 components can be stably projected to the own dataset (**b**) validating the biological origin of the differences and excluding a pure clustering because of confounding study-specific effects. Overall, this analysis shows that PCA heavily depends on the sample choice and that there are at least 6 biologically relevant (linear) dimensions in gene expression data.

One case that was already exemplified above is due to sample size effects, e.g. when the number of samples from a specific tissue is small compared to the overall number of samples (see Supplemental Discussion 1). This ratio can be increased by proper sub setting of the data, leading to the detection of new relevant dimensions that were hidden before, as exemplified by the subset analysis in the previous section (Fig. 4). Exploiting this sample size effect properly, it is even possible to select a subset of the Lukk dataset such that the first 6 PCs contain biological information (Fig. 5). However, while the strategy of looking at subsets can in principle reveal further dimensions, it is not always clear how to properly select these subsets in a purely unsupervised analysis. Furthermore, it is sometimes not straightforward to tell whether the detected dimensions contain relevant biological information or noise, unless an independent validation dataset is available. Therefore, we want to point the reader to another possibility to overcome these limitations.

**Figure 6:**
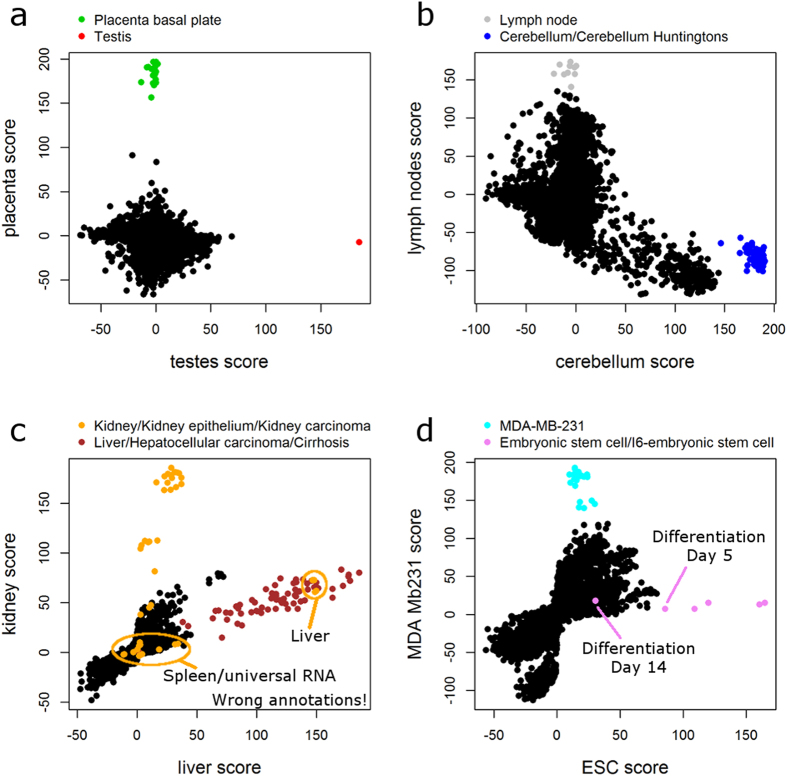
Using the PhysioSpace to project samples onto predefined directions with biological relevance. All samples of the Lukk dataset were projected onto the 93 tissue-specific expression patterns in the PhysioSpace, resulting in 93 scores per sample. Visualized are 8 of these scores, exemplifying the ability to detect additional clusters of samples that could only partially be detected by PCA. Color-coding is according to the sample annotation in the Lukk dataset. Some wrong annotations could be detected (see methods) explaining the unexpected behavior of the marked orange samples in (**c**) and the two “embryonic stem cell samples” in (**d**) that actually underwent 5 or 14 days of *in vitro* differentiation.

The idea here is to project the data onto predefined directions^7^. That is, we use well annotated publicly available datasets to construct a set of directions with clear biological meaning and project our data of interest onto these directions. One possibility of constructing such a set of directions is based on tissue specific expression patterns^7^. Using this approach, it is even possible to detect single samples that would usually be regarded as outliers, but which may represent a separate biologically relevant group, e.g. the single testis sample in the Lukk dataset (Fig. 6a). Some additional directions of this “PhysioSpace” are visualized in Fig. 6b–d, revealing further clusters of samples with clear biological meaning that were not identified by PCA.

The second case in which PCA fails to detect biological signals is due to a small effect size, meaning that the respective phenotype is associated with small overall differences in gene expression. In many cases, these phenotypic differences with a low overall effect size are not very stable, i.e. they are not well reproducible in independent studies^3^.

This statement can be explained by the observation that differential expression is typically not restricted to a few genes only^3^. Instead, we usually observe a continuum of differential expression, where the largest difference in a single gene is highly correlated with the overall effect size of this phenotypic difference. Thus, phenotypic differences with small effect sizes usually do not have very strong significances in individual genes making them almost indistinguishable from noise.

However, there are exceptions from this rule, which typically refer to genetic differences between the two groups. The simplest example of such an exception is the difference between male and female cells. This difference has a rather small effect size, but some genes with a very significant p-value (lying mainly on the Y-chromosome). Therefore, information on sex is often not contained in the first few PCs (at least in heterogeneous datasets), although it is very well reproducible. In Supplementary Fig. S4 we show this counter-example using a subset of the Lukk dataset that consists of all 208 B-cell lymphoma samples (90 female, 118 male) with available annotation of sex. Here, the first four PCs are not able to distinguish between male and female samples (Supplementary Fig. S4a). This is due to the small number of differentially expressed genes. In fact, mainly Y-chromosomal genes as well as the *XIST* gene, which lies on the X-Chromosome and is responsible for X-chromosome inactivation in females, are differentially expressed (Supplementary Fig. S4b).

In order to check in which dimension the information about sex differences is contained, we calculated the IR^3^ for increasing numbers of dimensions in the PCA space (Supplementary Fig. S4c). Here, we can observe that the IR stays above 0.7 when the dimension of the PCA space is increased up to 19 dimensions. Afterwards, the IR decreases, indicating that most of the sex information is contained in PCs 20 to 33.

Due to the small effect size, it is typically not possible to directly separate cells with genetic differences based on a PCA on gene expression data. Therefore, it is necessary to focus on different approaches. A relatively simple approach that can identify single genes that separate two groups is based on bimodality measures (Supplementary Fig. S4d). Using the kurtosis as a measure of bimodality (Teschendorff *et al*. 2006), we can detect two genes on the y chromosome, RPS4Y1 and DDX3Y, that have the lowest and third lowest kurtosis of all genes, respectively and that are able to separate male from female samples.

However, distinguishing between male and female cells is a rather straightforward task. It becomes more complicated when the genetic differences are more subtle or if they are present in a small subset of samples only. For these cases, more advanced approaches need to be used, such as the recently proposed “functional genomic mRNA profiling” (FGM) method^8^. The principle idea of this method is to remove all non-genetic biological variability in the data in order to emphasize the signal corresponding to genetic differences. This is achieved by first using PCA on very large datasets to identify typical sources of variation. All PCs fulfilling a certain reliability criterion are then divided into genetic or non-genetic components according to their autocorrelation over the genomic location. Based on this division into two groups it is then possible to correct only for non-genetic factors, revealing a much stronger signal of genomic differences in the residual expression matrix^8^.

## Discussion

Principal components analysis is frequently used in the analysis of whole-genome gene expression data, either for investigation of the overall structure of a dataset or for correction of confounding effects. Here, we focused on the former approach and investigated in which cases PCA fails to properly identify relevant biological information in the leading principle components. We distinguished between two different cases that are either due to a small effect size of the signal or due to a small number of samples containing the signal and gave examples of their practical relevance. It is important to note that the susceptibility of PCA to these two effects depends on the dataset under consideration. Very heterogeneous datasets with many different cell types or tissues, such as those analyzed by us, are strongly affected by these two effects, while more homogeneous datasets are generally less affected.

From a biological perspective, it seems to be helpful to distinguish between genetic and non-genetic biological signals. Purely genetic signals, e.g. copy number variations or sex differences, usually have a small effect size due to a limited number of affected genes and are therefore not represented in the leading PCs^8^. Non-genetic signals, e.g. environmental or cell fate differences, in contrast, typically affect a continuum of genes^3^ and are therefore well suited for PCA analysis in order to capture the correlation between genes, transforming the data into a lower dimensional representation. However, especially in the analysis of very large and heterogeneous datasets, it may happen that a certain cell type or environmental condition is represented by a small fraction of samples only. In these cases PCA also fails to properly separate these samples from all others and may therefore lead to wrong conclusions about the number of dimensions containing biological information in the dataset. In these cases, other methods such as the PhysioSpace approach^7^ can give further insights.

Based on our analyses, we cannot conclude on the actual linear intrinsic dimensionality of gene expression spaces, but we have shown that the linear dimensionality is definitely higher than previously suggested^4^. Fehrmann *et al*.^8^ even identified several hundred of so called “transcriptional components”, using measures of reliability (Cronbach’s alpha and split-half reliability) on their PCs to determine the number of reliable linear dimensions. They could show that all of these components are enriched for at least one predefined gene set, giving some indication of biological relevance despite of the well-known limitations of gene set enrichment approaches that assume independence of genes^9^. Another approach to distinguish between technical noise and actual biological information is based on control probes that are available on most array platforms^10^. Here it is assumed that the major technical variation is captured by control probes and can be identified and corrected for by performing a PCA on these control probes. A detailed investigation of this approach in the framework of gene expression microarray data and a comparison of different approaches to distinguish technical and biological information is, however, beyond the scope of the present article.

Estimation of intrinsic dimensionality is a complex task. The approaches discussed so far focus on linear dimension reduction. Thus, any nonlinearity in the actual intrinsic dimensions can lead to erroneous results and require appropriate nonlinear methods^11^.

In this respect it is also important to recognize that different cell or tissue types tend to cluster separately. Therefore, it is important to distinguish between the local (within cluster) and global (across cluster) dimensionality. The latter is especially problematic when considering nonlinear methods, since completely separate clusters can in principle be connected via a single nonlinear line. Therefore, clustered data make the notion of intrinsic dimensionality complicated on a global scale.

Another potential issue is that the analyzed datasets might not cover the full range of all possible gene expression patterns, e.g. due to sample selection or generally understudied tissues or cell types. This can also directly affect any method for estimation of intrinsic dimensionality.

From a more biological perspective, it is important to note that genes are connected to each other in a gene regulatory network, such that changes in the expression of one gene lead to changes in the expression of other genes. This restricts the set of states a network can potentially attain. Furthermore, many network states would not result in a viable cell, leading to additional restrictions. Therefore, it is biologically reasonable to assume that the intrinsic dimensionality of gene expression spaces is substantially lower than the number of genes in the dataset would allow. In fact, it has been observed that different perturbations of the gene regulatory network eventually converge to the same network state^12^. This network state can be regarded as an attractor of a complex dynamic system, explaining the observed clustering in the analyzed datasets.

In conclusion, we have refined the current understanding of the global structure of gene expression spaces, showed that the linear intrinsic dimensionality of gene expression spaces is higher than previously reported and explained the results of these reports by sample-size effects, limiting the interpretability of PCA results in terms of intrinsic dimensionality estimates. We hypothesize a structural difference between genetic and non-genetic biological signals and point to existing methods for both kinds of signals that overcome the described limitations of PCA.

## Materials and Methods

A graphical representation of the performed analyses, depicting interrelations, is provided in Supplementary Fig. S5. All figures can be reproduced using the R script available at http://www.combine.rwth-aachen.de/index.php/resources.html.

### Datasets and annotation

The Lukk dataset^4^, consisting of 5372 samples from the Affymetrix Human U133A microarray platform, and the corresponding sample annotation was downloaded in preprocessed form from ArrayExpress (http://www.ebi.ac.uk/arrayexpress/, accession number E-MTAB-62). The own dataset consisting of 7100 samples from the Affymetrix Human U133Plus 2.0 platform was compiled based on 108 public datasets from the Gene expression omnibus (GEO) database (http://www.ncbi.nlm.nih.gov/geo/, Supplemental Table S1). The raw data (CEL-files) were downloaded and preprocessed with Affymetrix Power Tools (http://www.affymetrix.com/estore/partners_programs/programs/developer/tools/powertools.affx) using the robust multi-array average (RMA) normalization method. The preprocessed dataset can be downloaded from http://www.combine.rwth-aachen.de/index.php/resources.html. The sample annotation was performed manually based on the description in the GEO database. Cancer cell lines and tissues were classified according to their primary tissue, leading to a less detailed distinction as in the Lukk dataset. This explains the reduced number of groups (192 instead of 369) in the own dataset compared to the Lukk dataset. In the latter, different cell lines and histologically different cancer tissues from the same primary site are classified into separate groups. The own dataset contains 213 *in vitro* (trans-) differentiated or teratoma samples which were not associated with any of the 192 groups.

### Comparison of PCA results

Principal components analysis of the Lukk and the own dataset were calculated in R version 3.1.2 using the *prcomp* function of the *stats* package. Subsequently, we compared the first three PCs of both datasets in order to determine whether they span similar spaces. For this analysis, we mapped the probes of the two platforms using the *getBM* function of the *biomaRt* package in R and performed linear regression analysis (R function *lm* of package *stats*) to explain the expression pattern of PCs 1 to 4 from the own dataset by the first three (first five, or first ten) PCs of the Lukk dataset. The resulting R^2^ values were reported as similarity measure of the two spaces.

The selection of a subset of samples from the own dataset with similar sample distribution as the Lukk dataset was performed in the following way. We used all 482 hematopoietic samples in the own dataset and randomly selected 74 brain, 163 cell line, 40 incompletely differentiated, 48 muscle, and 587 other samples. These numbers were chosen to match the proportion of samples in each of these large-scale groups to the respective proportion in the Lukk dataset.

The investigation of the effect of reduced numbers of liver or liver cancer samples in the own dataset was performed on the complete dataset with only the number of liver (cancer) samples reduced. Thus, we used all 6825 non-liver samples together with the specified number of liver (cancer) samples and performed a PCA on the dataset. PCs 1–3 did not change significantly with increasing number of liver samples (data not shown). Therefore, we focused on the differences in the liver-specific PC 4.

### Correlation analyses

The Pearson correlation of gene expression patterns between the 369 groups of the Lukk dataset as well as the 192 groups of the own dataset (Fig. 2a,d) was calculated between the vectors pointing from the overall mean of the entire dataset to the respective group mean. For the residual correlation after PCA-based decomposition (Fig. 2b,e), the residual vectors pointing from the three dimensional PCA space to the respective group means were used instead.

Within-group correlation was calculated in the same way between individual samples within one specific group. These calculations were performed for all groups that contain at least 10 samples. Depicted are the mean correlation values for each group (Fig. 2c,f).

### Information ratio

The information ratio is described in detail in^3^ and will be only briefly described here. The general idea is to decompose the expression data into two data matrices of the same size as the original data, one representing the projection onto the first three PCs and one representing the residual expression. For each of the two generated data matrices, the log-p-value between two groups, e.g. two different tissues, is calculated for each gene and plotted against the p-value of the original dataset. It is thus assessed which part of the expression difference between the two groups is captured by the first three PCs or the residual space, respectively. Strongly negative log-p-values are associated with high information content in the respective subspace. The p-values from the projected and residual space are then summarized into a single number ranging from 0 to 1, indicating whether most information is contained in the projected space (low IR values) or in the residual space (high IR values)^3^. This number can be interpreted as the proportion of group-specific information that is contained in the residual space.

### Analysis of sample subsets

For the analysis of sample subsets we also used the decomposition into the projected and residual data matrices. We then concentrated on the residual data matrix and performed a PCA on the respective subset of data, i.e. the cancer subset or the brain subset. PCA successively identifies the direction of largest variability in the space orthogonal to the already identified directions. Therefore, it would not make sense to perform PCA on the complete residual matrix, since this would exactly identify the fourth, fifth, and all further PCs from the original dataset. However, performing PCA on a subset of data can reveal different directions. In our case, the first two PCs of this subset analyses had a clear biological meaning (Fig. 4) as opposed to the fourth PC of the original complete dataset (Supplemental Fig. S1). Thus, performing PCA on subsets of data can reveal additional biologically relevant dimensions.

Validation of the biological relevance of the newly identified dimensions was then performed using our own dataset. As a first step, the own data was projected onto the first three PCs of the Lukk dataset (Supplementary Fig. S3, Fig. 4b). In order to do this, probesets of the two different microarray platforms were matched using the *biomaRt* package in R. Afterwards, the mean value for each gene (from our own dataset) was subtracted and the data were orthogonally projected onto the three PCs of the Lukk dataset using scalar products between the loading vector of each PC and the gene expression vectors. In a second step, we concentrated on subsets of the own dataset that correspond to the two subsets from the Lukk dataset, i.e. consisting of colorectal, liver, and ovarian cancer samples, as well as hypothalamus, cerebral cortex, and cerebellum samples. For both of these subsets the residual expression matrix was determined by subtracting the information that is contained in the three PCs of the Lukk dataset. Afterwards, the residual expression vectors were projected onto the respective first two “residual subset PCs” that were identified based on the subset analysis of the Lukk dataset (Fig. 4b insets).

### PhysioSpace analyses

For the PhysioSpace analyses the tissue-specific expression patterns that were determined based on the Human body index dataset (GEO accession GSE7307) were used^7^. Each individual sample of the Lukk dataset was compared to the overall mean of the Lukk dataset and the expression difference was projected onto the PhysioSpace as described in the original publication^7^. This results in 93 scores per sample that are associated with tissue specific expression. Eight of these scores were selected for visualization (Fig. 6) to exemplify the ability to detect additional clusters of samples that were partially not detectable by PCA.

Color-coding in Fig. 6 was performed according to the sample annotation provided by Lukk *et al*.^4^. We detected some samples that were annotated as “kidney”, but which showed a low “kidney score” in the PhysioSpace (Fig. 6c). In addition, some samples annotated as “kidney” seemed to be similar to liver. We then went back to the original source of these samples (GEO accession GSE2004) and detected that these samples were wrongly annotated in the Lukk dataset (Fig. 6c).

In a similar way, we could detect that two samples annotated as “embryonic stem cell” in the Lukk dataset actually underwent an *in vitro* differentiation for 5 or 14 days (ArrayExpress accession E-MEXP-303, Fig. 6d).

## Additional Information

**How to cite this article**: Lenz, M. *et al*. Principal components analysis and the reported low intrinsic dimensionality of gene expression microarray data. *Sci. Rep.* **6**, 25696; doi: 10.1038/srep25696 (2016).

## Supplementary Material

Supplementary Information

Supplementary Information

Supplementary Information
