# Supplementary material for: Principal components analysis and the reported low intrinsic dimensionality of gene expression microarray data
Source: Sci Rep. 2016 Jun 2;6:25696. doi: 10.1038/srep25696 (PMC4890592; doi:10.1038/srep25696)
Supplement: Supplementary Information [file srep25696-s1.pdf]

Principal components analysis and the reported low intrinsic dimensionality of gene expression microarray data

Michael Lenz,[1,2,3,*] Franz-Josef Müller,[4] Martin Zenke,[5,6] Andreas Schuppert,[1,2,*]

[1] Joint Research Center for Computational Biomedicine, RWTH Aachen University, 52062 Aachen, Germany

[2] Aachen Institute for Advanced Study in Computational Engineering Science (AICES), RWTH Aachen University, 52062 Aachen, Germany

[3] Maastricht Centre for Systems Biology (MaCSBio), Maastricht University, 6229 ER Maastricht, Netherlands

[4] Zentrum für Integrative Psychiatrie, University Hospital Schleswig-Holstein, Campus Kiel, Kiel, Germany

[5] Institute for Biomedical Engineering, Department of Cell Biology, RWTH Aachen University Medical School, 52074 Aachen, Germany

[6] Helmholtz-Institute for Biomedical Engineering, RWTH Aachen University, 52074 Aachen, Germany

Corresponding authors: Michael Lenz, Maastricht Centre for Systems Biology (MaCSBio), Maastricht University, Universiteitssingel 60, 6229 ER Maastricht, Netherlands, Tel. +31 (0) 43 38 81123, E-mail: michael.lenz@maastrichtuniversity.nl

Andreas Schuppert, Joint Research Center for Computational Biomedicine, RWTH Aachen University, Augustinerbach 2a, 52062 Aachen, Germany, Tel. +49 (0) 241 80 99202, E-mail: schuppert@aices.rwth-aachen.de

Supplemental Discussion 1

In order to explain the two cases in which PCA fails to detect biologically relevant information, we use the following illustrative statistical model to describe the gene expression vector $\vec{y_j}$ of sample j ($j = 1, \dots, n$):

$$\vec{y_j} = \sum_{i=1}^{d_b} a_{ij}\vec{u_i} + \sum_{l=1}^{d_c} c_{lj}\vec{w_l} + \sum_{k=1}^{d_n} b_{kj}\vec{v_k} + \vec{\varepsilon_j}. \qquad (1)$$

This simplified model consists of two qualitatively different types of biological signals (first and second term) and two different types of noise (third and fourth term). The first type of biological signal is associated with a binary coefficient a_{ij}, describing a certain group association, e.g. whether the sample is a heart tissue or not. The second type of biological signal is associated with a continuous coefficient c_{lj}, describing for instance the proliferation rate of the cells. The two types of noise can be distinguished in one term describing correlated noise (third term in the equation) and one describing uncorrelated noise ($\vec{\varepsilon_j}$), which is assumed to be independent identically distributed (i.i.d.) with variance σ^2 for each gene. All terms in the model are assumed to be pairwise orthogonal, i.e. the d_b biological signals of type one ($\vec{u_i}$), the d_c biological signals of type two $\vec{w_l}$, and the d_n correlated noise terms $\vec{v_k}$ are all assumed to be orthogonal to each other. The noise coefficient b_{kj} is a random variable with mean 0 and variance 1, the coefficient c_{lj} has also variance 1, and the coefficient a_{ij} is a binary variable describing a certain phenotype, where n_i samples are affiliated with this phenotype, having a value of 1, and $n - n_i$ samples are not affiliated with this phenotype, having a value of 0.

Since PCA detects directions of highest variability, a criterion for capturing noise instead of biological signal is that the noise variability is higher than the signal variability. Using our illustrative statistical model, we can determine the signal and noise variances in the limit of $n \to \infty$. The variances s^2 in direction of the two different signals are given by:

$$s^2 = \begin{cases} \frac{n_i}{n}\left(1 - \frac{n_i}{n}\right) ||\vec{u_i}||^2 + \sigma^2, & \text{signal type 1} \\ ||\vec{w_l}||^2 + \sigma^2 & \text{, signal type 2} \end{cases} \qquad (2)$$

The variances in the noise directions are given by

$$s^2 = \begin{cases} ||\vec{v_k}||^2 + \sigma^2 & \text{, noise type 1} \\ \sigma^2 & \text{, noise type 2} \end{cases} \qquad (3)$$

With this model, we can illustrate the two cases in which PCA fails to detect biological signals before capturing noise.

One case is due to sample size effects, i.e. due to small values of $\frac{n_i}{n}$ in the first row of equation (2). For instance, consider a typical cell type of the Lukk dataset with roughly $n_i = 15$ samples (5372/369). The variance corresponding to this tissue specific component can be calculated according to equation (2) to $0.0028||\vec{u_i}||^2 + \sigma^2$. The same component on a subset of the data with all 15 samples of this tissue and another 15 samples of other tissues would result in a variance of $0.25||\vec{u_i}||^2 + \sigma^2$ and would thus be almost 100 times higher (assuming that σ^2 is negligible). This effect explains why we can detect additional biologically relevant dimensions in subsets of the data (Fig. 4, Fig. 5).

The second case is due to a small effect size, i.e. a small value of $||\overrightarrow{u_i}||^2$ itself. In this case, even a perfect sample size of $n_i = \frac{n}{2}$ does not suffice to detect the biological signal in the leading PCs (Supplemental Fig. S4). Therefore, it is necessary to use different methods in order to detect these biological signals as described in the main text.

Supplemental Figures

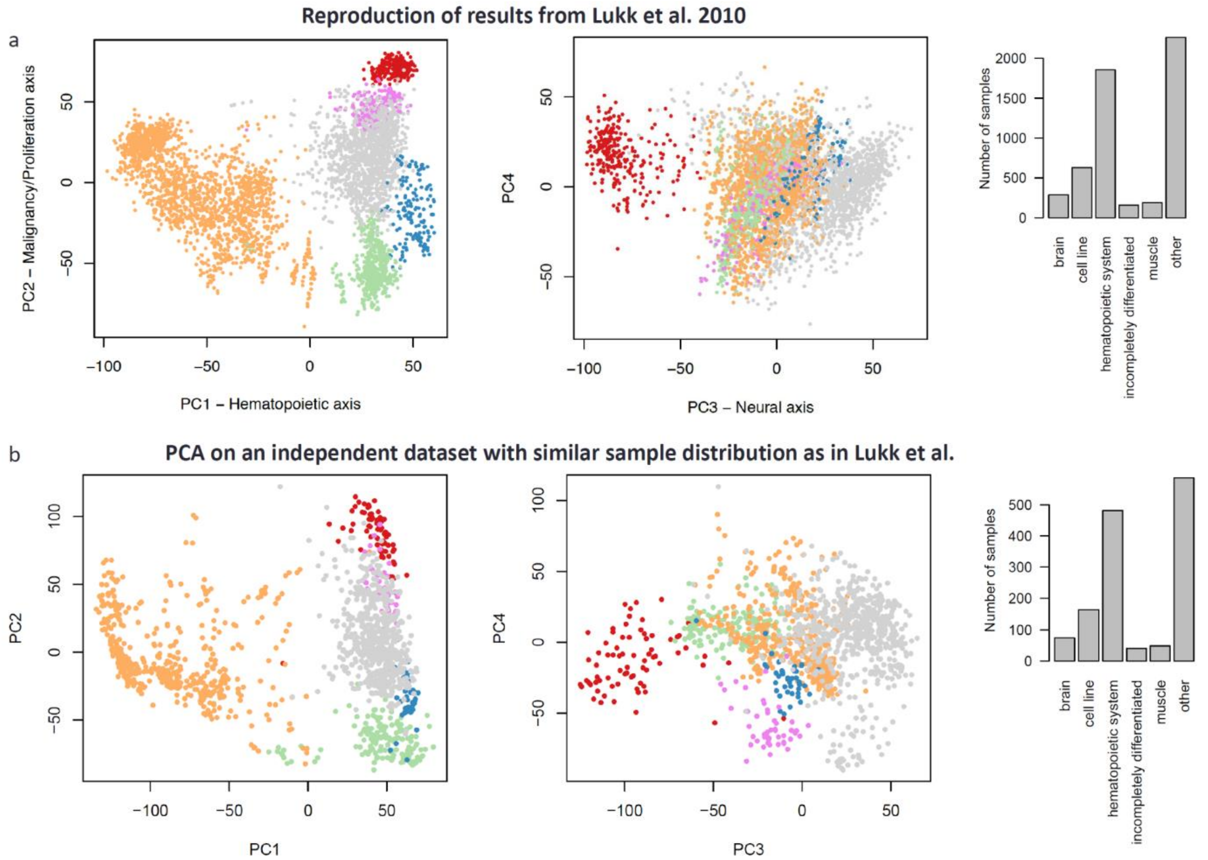

Supplemental Figure S1: Reproduction of PCA results from Lukk et al. and comparison to an independent dataset with similar sample distribution

(a) PCA was applied to the Lukk dataset, revealing associations with hematopoietic tissues, malignancy or proliferation, and brain tissues in the first three PCs (reproduced from [1]). The fourth PC is associated with an array quality metric [1]. The number of samples in each of the color-coded groups is depicted as barplot. (b) PCA applied to a subset of 1394 samples from the own dataset, with similar proportions of the color-coded groups as in the Lukk dataset (barplot). The results are surprisingly similar to those in (a) and differ substantially from those obtained with the complete own dataset (Fig. 1), indicating a strong sample-size effect.

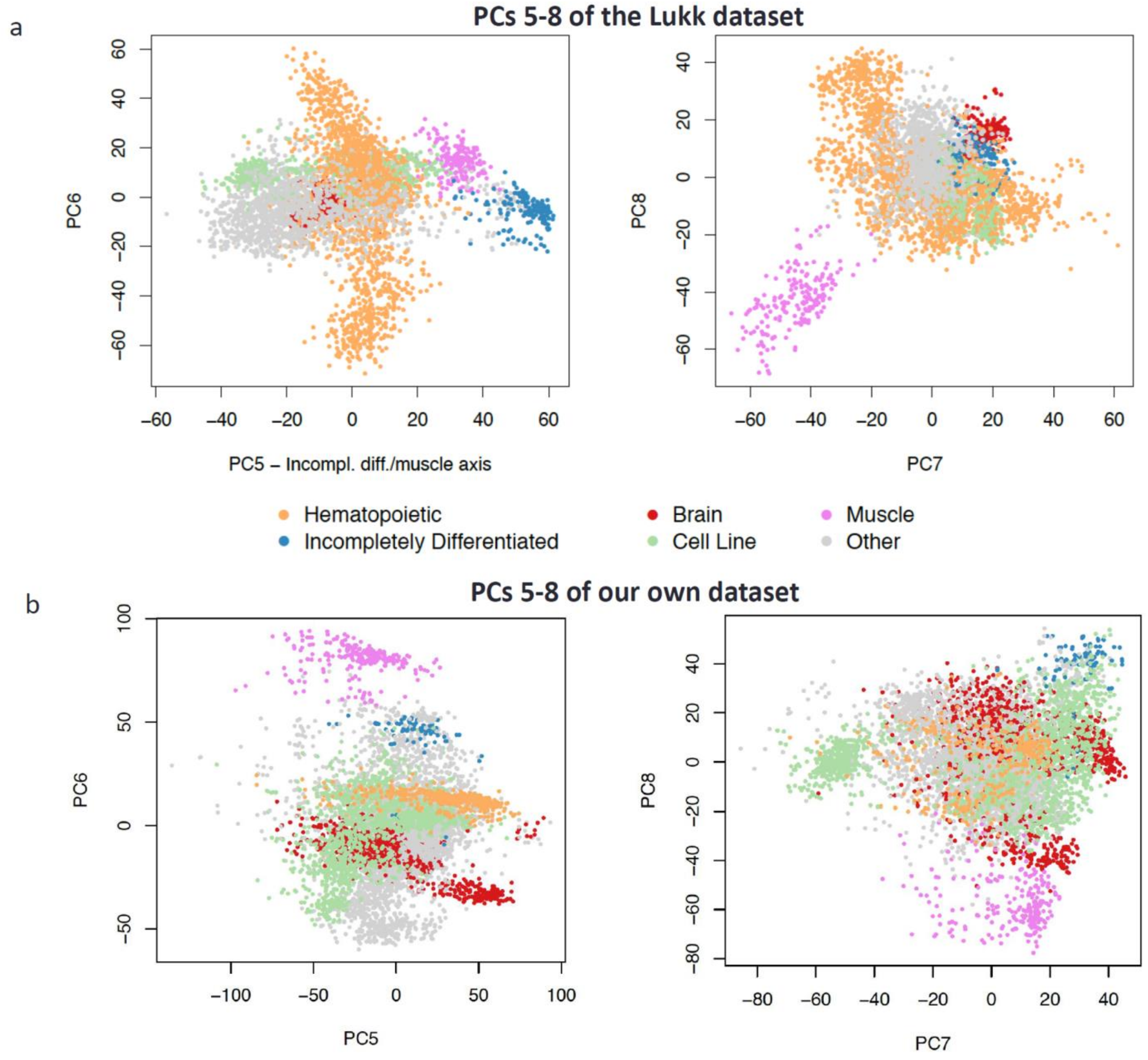

Supplemental Figure S2: Further principal components of both datasets

Principal components 5 to 8 of the Lukk dataset (a), and the own dataset (b). In the Lukk dataset, PC5 separates incompletely differentiated and, to a less extent, muscle tissues from the rest. PCs 7 and 8 separate muscle tissues from all others. PC5 of the own dataset has no clear biological meaning. PC6 and PC8 are specific for muscle tissues and PC7 partially separates pluripotent stem cells (green cluster on the left side) from the rest.

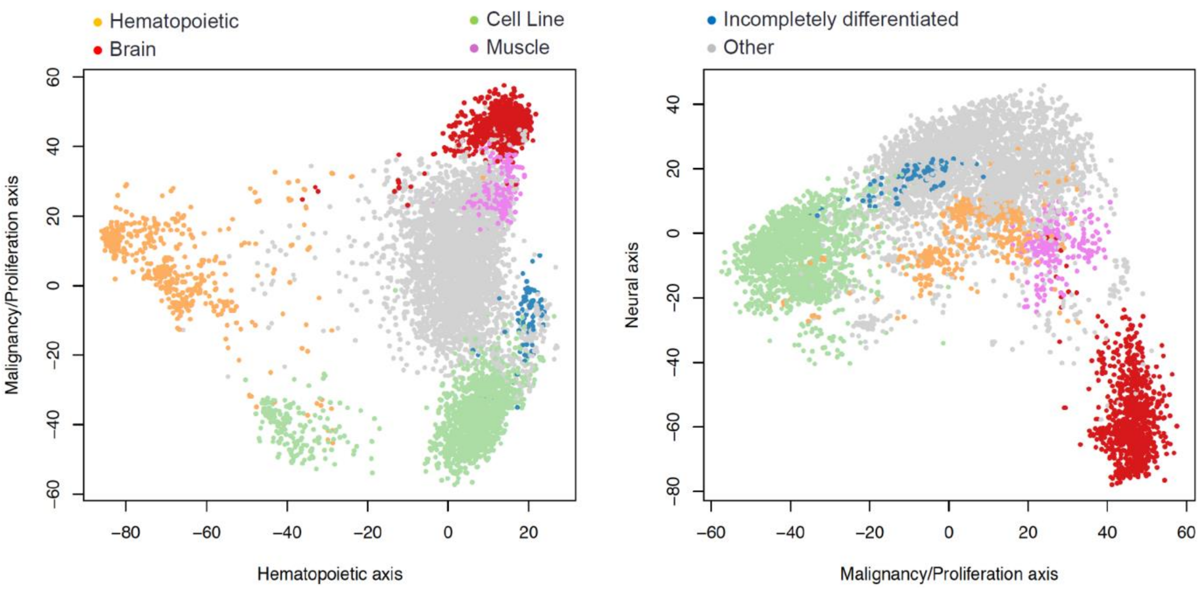

Supplemental Figure S3: Projection of the PCA-space to a different microarray platform

The PCA-space of the Lukk dataset (Affymetrix Human U133A array) was projected to the Affymetrix Human U133 Plus 2.0 platform. Visualized are the samples from the own dataset with color coding according to the large-scale groups as indicated. Both mappings reveal a clear separation of hematopoietic cells, brain tissues, and cell lines from all other samples with the same structure as in the Lukk dataset itself. This shows the possibility to robustly project this three dimensional PCA space to other Affymetrix arrays.

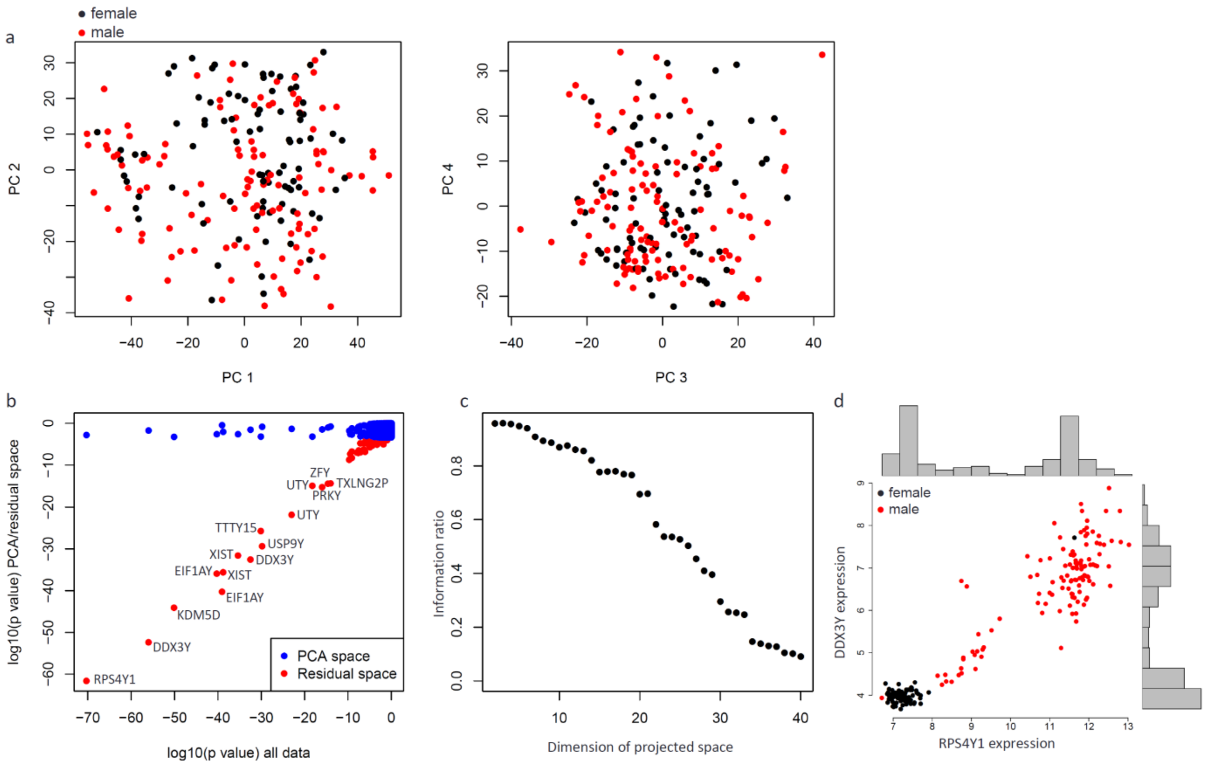

Supplemental Figure S4: Female and male B-cell lymphomas are hardly distinguishable based on PCA

All B-cell lymphomas from the Lukk dataset with available information on sex were subjected to PCA and information ratio analyses. (a) Visualized are the first 4 PCs, showing no distinction between male and female samples. (b) Analysis of differential expression in the projected (first 4 PCs) and residual space reveals that the complete information on sex is contained in the residual space. Differentially expressed genes lie mainly on the Y-chromosome. The sole exception is *XIST*, which lies on the X-chromosome, being responsible for X-chromosome inactivation in females. (c) Information ratio analyses with varying numbers of PCs in the projected space reveal that information on sex is mainly contained in PCs 20 to 33. (d) Unsupervised analysis of bimodal expression patterns can identify Y-chromosomal genes that are able to separate male and female samples.

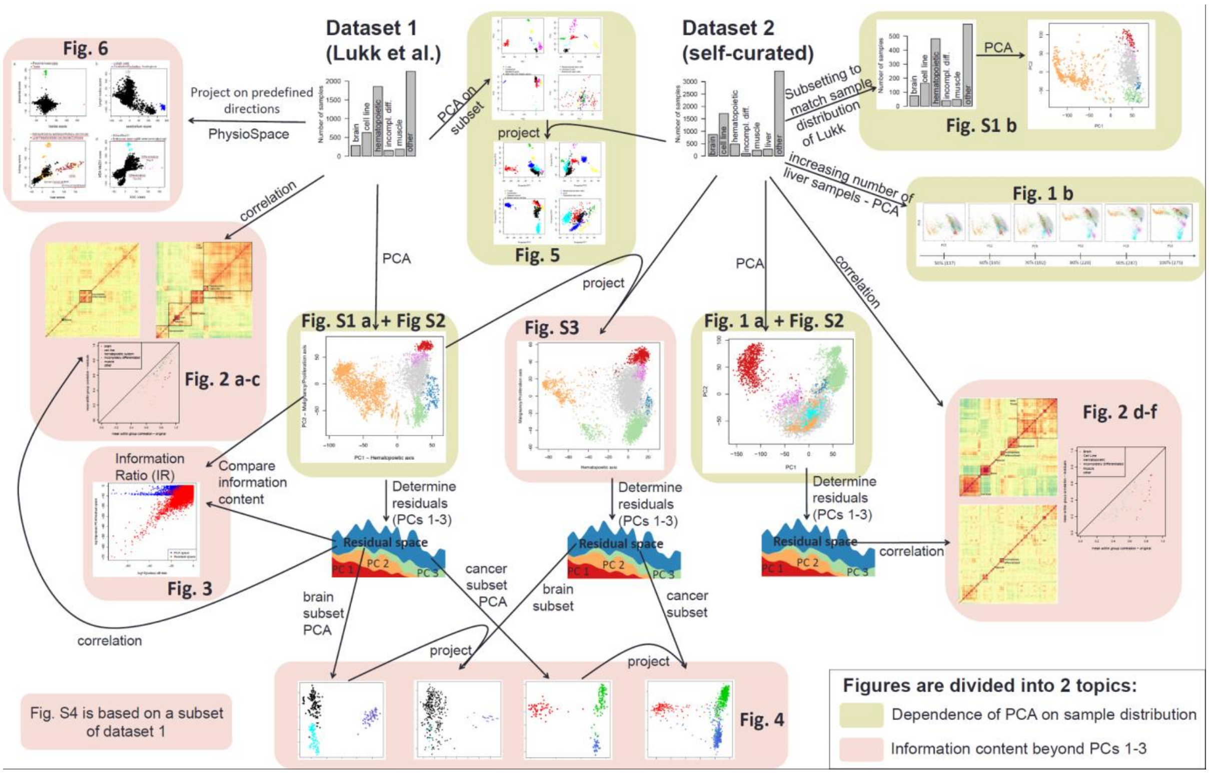

Supplemental Figure S5: Graphical overview of performed analyses

The steps taken to produce the article figures are illustrated in this graphics to support a detailed understanding of the performed analyses. The starting points for all analyses are two datasets, the Lukk and our own (self-curated) dataset. Furthermore, we use three different kinds of “residual datasets”, which were generated through a subtraction of the mean as well as the first three PCs (or projected PCs) from the original dataset. The “project” terms in the figure always link a PCA result and a dataset, giving rise to a new figure. This indicates that the (mean-subtracted) dataset is projected to the loading vectors of the indicated PCA result. This projection is used to validate the biological relevance and stability of detected dimensions on an independent dataset across microarray platforms.

Supplemental References:

1. Lukk, M., et al. A global map of human gene expression. *Nat. Biotechnol.* **28,** 322-324 (2010).
